# Determinants of Cervical Cancer Screening among Female Health Professionals in Harar Town, Eastern Ethiopia: A Cross-Sectional Study

**DOI:** 10.1155/2024/1430978

**Published:** 2024-06-08

**Authors:** Yahya Abdi Ziyad, Elias Jemal, Merga Dheresa, Ahmedin Aliyi Usso, Hassen Abdi Adem, Aboma Motuma, Mohammednur Abdo Komicha, Addis Eyeberu, Sherif Abdi Yuya

**Affiliations:** ^1^ School of Medicine College of Health and Medical Sciences Haramaya University, Harar, Ethiopia; ^2^ School of Nursing and Midwifery College of Health and Medical Sciences Haramaya University, Harar, Ethiopia; ^3^ School of Public Health College of Health and Medical Sciences Haramaya University, Harar, Ethiopia; ^4^ Department of Nursing Hiwot Fana Comprehensive University Hospital Haramaya University, Harar, Ethiopia; ^5^ Department of Anesthesia College of Medical and Health Sciences Dire Dawa University, Dire Dawa, Ethiopia

## Abstract

**Background:**

Early screening for cervical cancer is a key life-saving intervention in reducing maternal mortality and morbidity. Despite the high burden of cervical cancer, the coverage of cervical cancer screening is low in developing countries, including Ethiopia. There is a paucity of information on the utilization of cervical cancer screening among female health professionals in eastern Ethiopia. This study aimedto assess the determinants of cervical cancer screening among female health professionals in Harar town, eastern Ethiopia.

**Method:**

An institution-based cross-sectional study was conducted among 232 female health professionals in Harar town from September 01 to 30, 2022. Data were entered using EpiData version 3.1 and analyzed using SPSS version 27.0. Multivariable logistic regression analyses were conducted to identify significant factors for the level of cervical cancer screening. An adjusted odds ratio (AOR) with a 95% confidence interval was used to report the strength of association and statistical significance declared at *p* value < 0.05.

**Results:**

The prevalence of cervical cancer screening among female health professionals was 16.8% (95% CI: 11%, 22%). Higher education level (AOR = 4.28, 95% CI: 1.68, 10.90), use of contraceptives (AOR = 2.71, 95% CI: 1.17, 6.23), training on cervical cancer screening (AOR = 2.53, 95% CI: 1.05, 6.08), good knowledge about cervical cancer screening (AOR = 3.37, 95% CI: 1.44, 7.91), and positive attitude toward cervical cancer screening (AOR = 5.31, 95% CI: 2.04, 13.83) were independent factors that increased the utilization of cervical cancer screening.

**Conclusion:**

One in every six female health professionals was screened for cervical cancer. Education level, contraceptive use, cervical cancer screening training, cervical cancer screening knowledge, and attitude toward cervical cancer screening were the determinants of cervical cancer screening utilization among female health professionals. Improving the health professionals' knowledge and attitude toward cervical cancer screening through upgrading their education level and training on cervical cancer screening would be essential to improving the level of cervical cancer screening.

## 1. Introduction

Cervical cancer is a major public health problem and the fourth most common cancer affecting women globally. An estimated 604,000 new cases and 342,000 deaths of cervical cancer were reported each year worldwide, and 90% of these cases and deaths were from lower and middle-income countries (LMIC) [1, 2]. In sub-Saharan Africa (SSA), around 110,300 new cases and 50,000 death cases of cervical cancer are reported each year [3, 4]. The burden of cervical cancer-related maternal death is also high in Ethiopia, with a reported 7445 new cases and 4892 death cases each year in 2020 [5]. On the other hand, the incidence and mortality rate of cervical cancer among Ethiopian women were 26.4 and 18.4/100,000, respectively [6, 7]. The majority (80%) of women with cervical cancer presented with advanced disease [8].

Cervical cancer can develop after prolonged preinvasive lesions in the cervix [7]. Screening and early identification offer protective benefits by reducing the incidence and mortality of cervical cancer [9]. The World Health Organization (WHO), the United States Preventive Services Task Force (USPSTF), and the American Cancer Society (ACS) recommended that all eligible women should have screening for cervical cancer at least once every three years [10–12]. However, the absence of a national screening system and low access to the service have been reported to contribute to inefficient testing and late diagnosis and treatment [13]. It is mostly caused by human papillomavirus (HPV), specifically the two strains HPV 16 and HPV 18, which are responsible for about 70% of all cervical cancer cases worldwide [2]. The potential risk factors for HPV persistence and the development of cervical cancer are multiple sexual partners, sexually transmitted disease, long-term use of hormonal contraceptives, multiparity, early age of first sexual intercourse, early age at first birth, smoking, and immune suppression [14].

In countries with well-organized cervical screening programs, cervical cancer morbidity and death have declined. Despite the high burden of cervical cancer in developing countries, the coverage of cervical cancer screening is low in these countries [15]. The study indicated that the average coverage of cervical cancer screening for eligible women was 63% in developed countries, 19% in developing countries [16], 12.9% in SSA [17], and only 2.9% of eligible women in Ethiopia screened for cervical cancer [18, 19].

To avert the increasing morbidity and mortality associated with cervical cancer globally, the World Health Organization (WHO) and its partners have developed a triple-intervention strategy that seeks to ensure that by 2030, 90% of HPV vaccination coverage, 70% of women are screened twice at least in their lifetime, and 90% of women have access to cervical precancerous and cervical cancer treatment [20].

The Federal Minister of Health (FMOH) of Ethiopia has organized a national cancer control plan to be implemented from 2015 to 2020, which aims to reduce the cancer burden through primary prevention, lifestyle change, screening, and early diagnosis to reach 80% of screening coverage through conducting community awareness on the availability, importance of screening, and treatment. Despite the high burden of cervical cancer in developing countries, including Ethiopia, the level of cervical cancer screening utilization was low [16–19, 21]. The data shows the level of cervical cancer screening among female health professionals is limited in eastern Ethiopia. Female health professionals play a major role in creating awareness and promoting cervical cancer screening among the population they are serving. Practicing cervical cancer screening by female health professionals is often crucial to gaining women's confidence. Identifying the associated factors of the utilization of cervical cancer screening among female health professionals is important to improve overall screening coverage.

In addition, the WHO 90–70–90 triple-intervention strategies by 2030 [20], the goal of the Strategic Plan for Cervical Cancer Prevention and Control, would be achieved by improving the utilization of cervical cancer screening among female health professionals [22]. Overall, there is a scarcity of information on cervical cancer screening among female health professionals in eastern Ethiopia. Therefore, this study aimed to assess the determinants of cervical cancer screening among female health professionals in Harar town, eastern Ethiopia.

## 2. Materials and Methods

### 2.1. Study Design and Setting

An institution-based cross-sectional study was conducted in Harar town in eastern Ethiopia from 01 to 30 September 2022. Harar town is the capital of Harari Regional State, located 526 kilometers east of Addis Ababa (the capital of Ethiopia). The town has a total population of 139,380; 68,048 are females, and 43,339 are women in the reproductive age group. According to the Harari Regional Health Bureau's 2022 annual report, there are three public hospitals (two government and one private), eight public health centers, and 20 private clinics in the town, with 1800 health professionals and 523 female health professionals.

Among the public health facilities in Harar town, two public hospitals (Hiwot Fana Comprehensive Specialized University Hospital and Jegol General Hospital) and two health centers (Janela Health Center and Aboker Health Center) have screening centers for cervical cancer. According to the national standard, cervical screening procedures are performed by health professionals who have demonstrated competency: nurses, midwives, health officers, general practitioners, residents, and specialists [23].

### 2.2. Population

All female health professionals in public health facilities in Harar town were the source population. Female health professionals who were working in selected public health facilities in Harar town were the study population. All female health professionals aged 30 years and older who worked in selected health facilities for at least six months were included, while those who were critically ill and those who were on leave of any type, including training, education, and fieldwork activities during the data collection period, were excluded from the study.

### 2.3. Sample Size Determination and Sampling Procedure

The sample size (*n* = 232) was computed using Epi Info version 7.1, a single-population proportion formula with the following assumptions: a confidence level of 95%; a margin of error of 5%; a proportion of cervical cancer screening (10.2%) in Mekelle, northern Ethiopia, using a design effect of 1.5; and a 10% nonresponse rate [6].

Study participants were selected using a multistage stratified sampling technique. First, public health facilities were stratified into hospitals and health centers. All public health facilities in Harar town that have screening centers for cervical cancer (two hospitals and two health centers) were selected purposively. Then, the estimated sample size was proportionally allocated to selected health facilities based on the number of registered female health professionals in the facility during the last six months before the data collection. We prepared a separate sampling frame for each health facility using their actual numbers of permanently hired female health professionals in 2022. The eligible participants were recruited using a systematic sampling technique.

### 2.4. Data Collection Tools and Measurements

Pretested-structured questionnaires adapted from relevant published literature [6, 21, 22, 24–30] were used to collect data from participants through a self-administered interview conducted over a month. The questionnaire contains information on the sociodemographic characteristics of the participants, reproductive factors, knowledge and attitudes toward cervical cancer screening, and level of cervical cancer screening utilization.

#### 2.4.1. Female Health Professionals

Female health workers are those who provide treatment, care, and advice to patients/clients based on formal training, including nurses, doctors, health officers, lab technicians/technologists, pharmacists/druggists, anesthetists, radiographers, midwives, and psychiatrists [24, 31].

#### 2.4.2. Cervical Cancer Screening Utilization (CCS)

It was considered “yes” when participants have been screened at least once for cervical cancer in their lifetime and “no” unless otherwise [24, 32, 33].

#### 2.4.3. Knowledge about Cervical Cancer Screening

It was measured by seven questions asking about knowledge about cervical cancer screening, and each item was coded “1” when responding “yes” to at least one correct answer and coded “0” when responding “no” to all correct answers; then, a composite index score was computed from seven items ranging from 0 to 7, and the mean was calculated from a total score. The female health professionals who scored mean and above were considered to have good knowledge, and those who scored less than the mean were considered to have poor knowledge [6, 7, 26, 34].

#### 2.4.4. Attitude to Cervical Cancer Screening

It was measured using eight Likert scale items, and each item was rated “1” when responding strongly disagree, “2” disagree, “3” indifferent, “4” agree, and “5” strongly agree. Then, the composite index score was computed from six items, and the female health professionals had a positive attitude when scored at the mean and above and a negative attitude when scored less than the mean [6, 32, 34].

### 2.5. Data Quality Control

Data quality was maintained using questionnaires adapted from relevant published literature and contextualized to the study purpose and setting. The questionnaire first prepared in English was translated into local languages (Afan Oromo and Amharic) and back to English by two experts with good command of both languages. We pretested an adapted questionnaire on 5% of the total sample (12) to check their validity in a separate nonselected facility in the town, and changes were made accordingly. Four data collectors collected the data under the supervision of two supervisors after training for one day on the objective of the study and the data collection technique.

### 2.6. Statistical Analysis

After checking for completeness, the data were entered using EpiData version 3.1 and analyzed using SPSS version 27.0. Descriptive statistics, frequency, measures of central tendency, and dispersion were used to characterize the participants and assess the levels of cervical cancer screening. Before analysis, the internal consistencies of items were checked for each composite index score using reliability analysis (Cronbach's *α*). We observed the internal consistency of the composite indexes in the knowledge of cervical cancer items (Cronbach's *α* = 0.85) and attitude toward cervical cancer screening items (Cronbach's *α* = 0.86). Bivariable and multivariable logistic regression analyses were employed to identify factors associated with cervical cancer screening utilization. In multivariable logistic regression, work experience, qualification, level of education, history of giving birth, use of contraceptives, training on cervical cancer screening, multiple sexual partners, treatment of sexually transmitted infections, knowledge of cervical cancer screening, and attitude toward cervical cancer screening variables were included in the model. Overall, model adequacy was confirmed using the Hosmer and Lemeshow goodness-of-fit test at a *p* value > 0.05. We ruled out and confirmed the absence of numerical errors and multicollinearity in the model (each predictor-standardized residual was less than the absolute value of three, Cook's distance was less than the absolute value of one, and the standard error of each coefficient (*β*) was less than two). An adjusted odds ratio (AOR) with a 95% confidence interval was used to report association and significance declared at a *p* value < 0.05.

## 3. Results

### 3.1. Characteristics of Participants

A total of 220 (95%) female health professionals participated in the study. The mean ± SD age of the participants was 41.08 ± 10, and the majority (64.5%) of the participants were less than or equal to 40 years old. One hundred forty-one (64.1%) of the participants were married, about 141 (64.1%) were nurses by profession, 120 (54.5%) were qualified at diploma level, and the majority (718%) of the participants had less than ten years of working experience (Table 1).

One hundred seventy-one (77%) respondents have been sexually active, and the majority (53.5%) of them started their first sexual intercourse as teenagers. Slightly more than half (53.2%) of the participants have ever given birth, and the majority (57.7%) of the participants used at least one type of contraceptive method. About 69 (31.4%) of the participants had multiple sexual partners, and 25 (11.4%) of them were also treated for sexually transmitted diseases. The majority (78.2%) of participants have never received training on cervical cancer screening. Ninety-seven (44.1%) of participants had good knowledge of cervical cancer screening. Among attitudes toward cervical cancer screening, 102 (46.4%) participants had positive attitudes (Table 2).

### 3.2. Utilization of Cervical Cancer Screening

This study revealed that the prevalence of cervical cancer screening utilization was 16.8% (95% CI: 12%, 22%) among female health professionals in the study area. Based on health professions, the majority (40.0%) of female health workers utilized cervical cancer screening, followed by psychiatrists (33.3%), physicians (22.2%), midwives (18.4%), pharmacists (16.7%), nurses (16.5%), and laboratories (14.3%). The major reasons reported for not using cervical cancer screening were being busy and lack of time for screening (18.2%), feeling healthy (16.4%), lack of information (13.6%), being afraid of the test (12.3%), feeling shyness (10.9%), and fear of positive result (11.8%).

### 3.3. Determinants of Cervical Cancer Screening

The bivariable analysis showed that work experience, qualification level of education, history of giving birth, use of contraceptives, training on cervical cancer screening, multiple sexual partners, treatment of sexually transmitted infections, knowledge of cervical cancer screening, and attitude toward cervical cancer screening were determinant factors of cervical cancer screening among female health professionals. However, in multivariable logistic regression analysis, education level, use of contraceptives, training on cervical cancer screening, knowledge of cervical cancer screening, and attitude toward cervical cancer screening were independent factors associated with the utilization of cervical cancer screening among female health professionals.

Female health professionals who have degrees and above the education level were 3.6 times more likely (AOR = 3.64, 95% CI: 1.45, 9.15) to utilize cervical cancer screening compared to those who have diploma qualifications. The female health professionals who had ever used contraceptive methods were about 2.5 times (AOR = 2.72, 95% CI: 1.16, 6.40) more likely to utilize cervical cancer screening compared to those who had never used contraceptives. Those who received cervical cancer screening training were 2.7 times more likely to be screened compared to their counterparts (AOR = 2.72, 95% CI: 1.16, 6.40). Female health professionals with good knowledge of cervical cancer screening were 3.3 times more likely to utilize cervical cancer screening (AOR = 3.31, 95% CI: 1.40, 7.81) than those with poor knowledge. The participants with a positive attitude toward cervical cancer screening were 4.6 times (AOR = 4.63, 95% CI: 1.78, 12.02) more likely to utilize cervical cancer screening than those with a negative attitude (Table 3).

## 4. Discussion

This study investigated the utilization and associated factors of cervical cancer screening among female health professionals in Harar town, eastern Ethiopia. The findings of this study revealed one in every six female health professionals screened for cervical cancer in Harar town. Level of education qualification, use of contraceptive methods, training on cervical cancer screening, knowledge of cervical cancer screening, and attitude toward cervical cancer screening were independent predictors of cervical cancer screening utilization among female health professionals.

The finding of this study indicated that 16.8% of female health professionals screened for cervical cancer at least once in their lives, which is consistent with the studies conducted in Ethiopia (17.0%) [35], southern Ethiopia (19.6%) [31], Uganda (19.0%) [36], India (11.9%) [37], and Korea (13.0%) [38]. However, this finding was higher than the studies conducted in southern Ethiopia (11.4%) [7]; Arba Minch, southern Ethiopia (9.6%) [24]; Mekelle, northern Ethiopia (10.7%) [33]; Sokoto, Nigeria (10.0%) [39]; Ghana (11.6%) [40]; Haiti (4.0%) [41]; and rural India (7.0%) [42]. This difference might be due to sociodemographic differences; only one type of professional (nurses) was included in the study conducted in Mekelle, northern Ethiopia [33], while all health professionals were included in our study. In addition, the possibility for this variation might be due to the difference in the level of education: 70% of female health professionals had diploma qualifications [7], compared to 54% in our study. In addition, this difference might be due to the methodological differences that we included hospitals and health centers, while a comparable study conducted in Nigeria was among one type of health facility [39].

On the other hand, this finding was lower than the studies conducted in southwest Nigeria (57.6%) [43]; Osun State, Nigeria (75.3%) [44]; Ibadan, Nigeria (32.6%) [45]; Japan (24.0%) [46]; and Brazil (94.7%) [47]. These differences might be due to different levels of participants' knowledge and attitudes toward cervical cancer screening. For instance, around 93% of female professionals were knowledgeable about cervical cancer in Brazil, while 44% were in our study area. The study area is also another possible explanation for this difference. These might be related to the fact that the quality of health professionals and healthcare systems is better in developed countries.

This study revealed that the qualification level of education was a significant factor in the utilization of cervical cancer screening. Female health professionals who have degrees and above were four times more likely to utilize cervical cancer screening than those who have diplomas. This finding was supported by studies conducted in Hossana, southern Ethiopia [31]; Wolaita, southern Ethiopia [48]; and Nigeria [39]. This implies that as the female health professional's qualification level of education improves, they become more knowledgeable of the causes, risk factors, prevention, screening methods of cervical cancer, and the importance of routine cervical cancer screening. This indicates that the presence of opportunities to improve the qualification level of education to a degree and above is important in improving the utilization of cervical cancer screening among female health professionals.

The use of contraceptive methods was two times more likely to increase the utilization of cervical cancer screening among female health professionals. This finding was supported by previous studies conducted indicating that women who utilized reproductive health services were more likely to utilize cervical cancer screening. In addition, women who have access to modern contraceptive methods through attending health facilities were more likely to get information about the advantages of early detection of cervical cancer [22, 49].

The odds of utilizing cervical cancer screening were higher among female health professionals who received cervical cancer screening training than those who did not receive training. This result indicated that female health professionals who received training on cervical cancer screening would have understood the screening procedure and the importance of early detection and treatment of cervical cancer.

In addition, female health professionals who had good knowledge about cervical cancer screening were three times more likely to utilize cervical cancer screening compared to those who had poor knowledge. This finding was supported by studies conducted in Hossana, southern Ethiopia [31], and Arba Minch, southern Ethiopia [24], which indicated that female health professionals' knowledge status has a significant effect on the utilization of cervical cancer screening.

Furthermore, female health professionals' attitude toward cervical cancer screening was a significant predictor of cervical cancer screening utilization. Having a positive attitude toward cervical cancer screening was five times more likely to increase the utilization of cervical cancer screening by female health professionals. This finding was supported by studies conducted in Arba Minch, southern Ethiopia [50], and in Debre Markos, northwest Ethiopia [34].

The strength of this study was that it was conducted among health service providers, which is uncommon in Ethiopia. A self-administered questionnaire was used to assess the utilization of cervical cancer screening and its associated factors, which has reduced social disparities and the problem of recall bias. The study was conducted among female health professionals in public health facilities, which may fail to generalize among female health professionals in the private sector, which was the limitation of the study.

## 5. Conclusions

This study showed that the proportion of cervical cancer screening among female health professionals was low. Improving the health professionals' knowledge and attitude toward cervical cancer screening through upgrading their education level and providing training on cervical cancer screening would be needed to increase the level of cervical cancer screening. In addition, encouraging female health professionals to utilize modern contraceptives was essential to improving the uptake of cervical cancer screening.

## Figures and Tables

**Table 1:** Sociodemographic characteristics of female health professionals in Harar town, eastern Ethiopia, 2022 (*n* = 220).

Characteristic	Frequency	Percent
Age (complete year)		
≤40	142	64.5
>40	78	35.5
Marital status		
Single	68	30.9
Married	141	64.1
Others^∗^	11	5
Profession		
Midwives	38	17.3
Nurses	141	64.1
Physician	9	4.1
Health officer	5	2.3
Laboratory	14	6.4
Pharmacy	18	8.2
Anesthetists	4	1.8
Radiographer	8	3.6
Psychiatrists	3	1.4
Qualification level of education		
Diploma	120	54.5
BSc and above	100	45.5
Department		
Obstetrics/gynaecology	72	32.7
Internal medicine	39	17.7
Surgery	22	10.0
Pediatrics	37	16.8
Outpatient department	10	4.5
Laboratory	14	6.4
Pharmacy	17	7.7
Operation room	9	4.1
Year of experience		
<10 years	158	71.8
≥10 years	62	28.2
Monthly income in ETB		
2000-4000	21	9.5
4000-12000	184	83.6
>12000	15	6.8

ETB: Ethiopian birr. ^∗^Divorced or widowed.

**Table 2:** Reproductive and knowledge-related characteristics of female health professionals in Harar town, eastern Ethiopia, 2022 (*n* = 220).

Characteristic	Frequency	Percent
Ever practiced sexual intercourse		
Yes	171	77
No	49	23
Age at 1^st^ sexual intercourse		
<20	92	41.8
≥20	79	35.9
Never practiced	49	22.3
Ever gave birth		
Yes	117	53.2
No	103	46.8
Multiple sexual partners		
Yes	69	31.4
No	151	68.6
Ever treated for sexually transmitted infection		
Yes	25	11.4
No	195	88.6
Ever used contraceptive		
Yes	90	40.9
No	130	59.1
Received training on cervical cancer screening		
Yes	48	21.8
No	172	78.2
Know the route of cervical cancer transmission		
Yes	184	83.6
No	36	16.4
Know the risk factors for cervical cancer		
Yes	121	55.0
No	99	45.0
Know the signs and/or symptoms of cervical cancer		
Yes	91	41.4
No	129	58.6
Know methods of cervical cancer prevention		
Yes	108	49.1
No	112	50.9
Know the methods of cervical cancer screening		
Yes	105	47.7
No	115	52.3
Know the recommended age for cervical cancer screening		
Yes	194	88.2
No	26	11.8
Know the types of cervical cancer treatment		
Yes	85	38.6
No	135	61.4
Overall knowledge about cervical cancer		
Good knowledge	97	44.1
Poor knowledge	123	55.9
Attitude toward cervical cancer screening		
Positive	102	46.4
Negative	118	53.6

**Table 3:** Determinants of cervical cancer screening among female health professionals in Harar town, eastern Ethiopia, 2020 (*n* = 220).

Variables	Screened cervical CA		cOR (95% CI)	aOR (95% CI)
	Yes, *n* (%)	No, *n* (%)		
Work experience in the year				
<10	24 (15.2)	134 (84.8)	1	1
≥10	13 (21.0)	49 (79.0)	1.48 (0.70, 3.14)	1.10 (0.44, 2.73)
Education qualification				
Diploma	15 (12.5)	105 (87.5)	1	1
Degree and above	22 (11.7)	78 (78.0)	1.97 (0.96, 4.05)	3.64 (1.45, 9.15)^∗∗^
Ever gave birth				
Yes	25 (21.4)	86 (78.6)	2.06 (0.98, 4.35)	2.01 (0.84, 4.82)
No	12 (11.7)	97 (88.3)	1	1
Ever used contraceptive				
Yes	24 (26.7)	98 (77.3)	3.27 (1.56, 6.85)^∗∗^	2.46 (1.08, 5.60)^∗^
No	13 (10.0)	85 (90.0)	1	1
Trained cervical cancer screening				
Yes	18 (37.5)	30 (62.5)	4.83 (2.27, 10.27)^∗∗∗^	2.72 (1.16, 6.40)^∗∗^
No	19 (11.0)	153 (89.0)	1	1
Multiple sexual partners				
Yes	14 (20.3)	55 (79.7)	1.42 (0.68, 2.96)	1.51 (0.64, 3.58)
No	23 (15.2)	128 (84.8)	1	1
Ever treated STI				
Yes	7 (28.0)	18 (72.0)	2.14 (0.82, 5.56)	2.20 (071, 6.82)
No	30 (15.4)	165 (84.6)	1	1
Cervical CA screening knowledge				
Good knowledge	27 (16.3)	70 (72.2)	4.36 (1.99, 9.55)^∗∗∗^	3.31 (1.40, 7.81)^∗∗∗^
Poor knowledge	10 (8.1)	113 (91.9)	1	1
Attitude toward CA screening				
Positive	26 (25.5)	76 (74.5)	3.33 (1.55, 7.14)^∗∗^	4.63 (1.78, 12.02)^∗∗∗^
Negative	11 (9.3)	107 (90.7)	1	1

^∗^ *p* value < 0.05; ^∗∗^*p* value < 0.01; ^∗∗∗^*p* value < 0.001. aOR: adjusted odds ratio; CA: cancer; STI: sexual transmitted infection.

## Data Availability

The data of this study are presented in the main manuscript. Any additional files (data) that support the findings are available from the corresponding author upon reasonable request.

## Abbreviations

AOR: Adjusted odds ratio
CCS: Cervical cancer screening
LMIC: Lower and middle-income countries
SSA: Sub-Saharan Africa
WHO: World Health Organization
VIA: Visual inspection of the cervix with acetic acid.
